# ﻿Three new spider species of *Belisana* Thorell, 1898 (Araneae, Pholcidae) from karst caves, with a list of *Belisana* species from Guangxi, China

**DOI:** 10.3897/zookeys.1209.127951

**Published:** 2024-08-14

**Authors:** Ludan Zhang, Ying Wang, Shuqiang Li, Xiaoqing Zhang, Zhiyuan Yao

**Affiliations:** 1 College of Life Science, Shenyang Normal University, Shenyang 110034, Liaoning, China Shenyang Normal University Shenyang China; 2 Institute of Zoology, Chinese Academy of Sciences, Beijing 100101, China Institute of Zoology, Chinese Academy of Sciences Beijing China

**Keywords:** Biodiversity, cellar spiders, checklist, invertebrate, morphology, new species, taxonomy

## Abstract

Three new species of the genus *Belisana* Thorell, 1898 are described from karst caves in Guangxi, China: *Belisanalangping* Zhang, Li & Yao, **sp. nov.** (♂♀), *B.lingui* Zhang, Li & Yao, **sp. nov.** (♂♀), and *B.tianyang* Zhang, Li & Yao, **sp. nov.** (♂♀). In addition, a list of all *Belisana* species from Guangxi is also provided.

## ﻿Introduction

*Belisana* Thorell, 1898, the second largest genus in Pholcidae, includes 157 species (WSC 2024). These species occupy a variety of micro-habitats, e.g., under rocks, in caves, on the underside of leaves, among leaf litter, and amidst foliage in the canopy (Huber 2005; Yao et al. 2015; Zhao et al. 2023a). They are distributed mainly in southern China, as well as in the Indo-Malayan and Australasian regions (Huber 2005; Yao et al. 2013, 2018; Zhu et al. 2020a; Zhu and Li 2021). Southern China exhibits remarkable diversity of this genus, with 71 species (45%) recorded to date. Within southern China, the species count from Yunnan (31 spp.) far outstrips those of Hainan (10 spp.), Guangxi (8 spp.), Guizhou (8 spp.), and Tibet (7 spp.) (Zhang and Peng 2011; Zhu et al. 2020a, b; Zhang et al. 2024). Furthermore, in Fujian, Guangdong and four other provinces, only seven species have been recorded (Zhu et al. 2020a). Recently, a series of surveys of pholcid spiders have been undertaken in China and a large number of new species have been reported (e.g., Yao et al. 2021; Lu et al. 2022; Zhao et al. 2023b; Yang et al. 2024a, b). Nevertheless, these efforts focused on *Pholcus* Walckenaer, 1805 from northern and central China, with relatively few reports on *Belisana* from southern China (Yang et al. 2023; Zhao et al. 2023a; Wang et al. 2024; Zhang et al. 2024).

**Figure 1.**
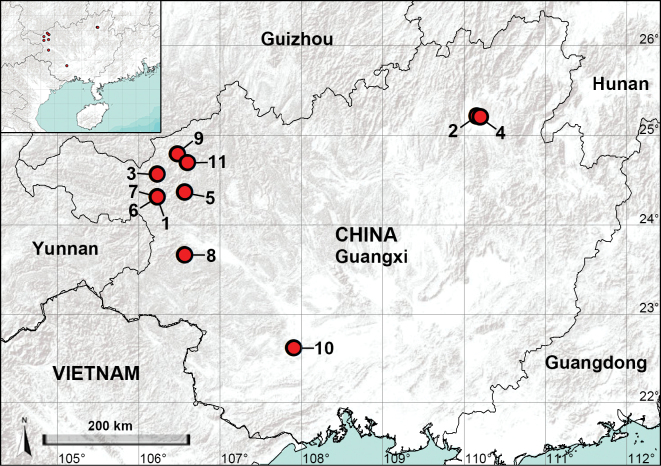
Distribution records of the *Belisana* species from Guangxi, China **1***Belisanacolubrina* Zhang & Peng, 2011 **2***B.guilin* Yao & Li, 2020 **3***B.langping* sp. nov. **4***B.lingui* sp. nov. **5***B.naling* Yao & Li, 2020 **6***B.parallelica* Zhang & Peng, 2011 **7***B.tianlinensis* Zhang & Peng, 2011 **8***B.tianyang* sp. nov. **9***B.tongle* Zhang, Chen & Zhu, 2008 **10***B.xuanguan* Yao & Li, 2020 **11***B.zhangi* Tong & Li, 2007.

Guangxi is located in the southwest of China. The karst landform is widely distributed in the northern part of Guangxi. The aim of this work is to describe three new *Belisana* species from karst caves in northern Guangxi (Fig. 1) and provide a list of the species of this genus from Guangxi (Table 1).

**Table 1.** A list of all *Belisana* species from Guangxi, China.

Species	Habitat	Reference
*B.colubrina* Zhang & Peng, 2011	/	Zhang and Peng (2011)
*B.guilin* Yao & Li, 2020	karst cave	Zhu et al. (2020a)
*B.langping* sp. nov.	karst cave	this paper
*B.lingui* sp. nov.	karst cave	this paper
*B.naling* Yao & Li, 2020	karst cave	Zhu et al. (2020a)
*B.parallelica* Zhang & Peng, 2011	/	Zhang and Peng (2011)
*B.tianlinensis* Zhang & Peng, 2011	/	Zhang and Peng (2011)
*B.tianyang* sp. nov.	karst cave	this paper
*B.tongle* Zhang, Chen & Zhu, 2008	karst cave	Zhang et al. (2008)
*B.xuanguan* Yao & Li, 2020	karst cave	Zhu et al. (2020a)
*B.zhangi* Tong & Li, 2007	karst cave	Tong and Li (2007)

## ﻿Materials and methods

Specimens were examined and measured with a Leica M205 C stereomicroscope. Left male palps were photographed. Epigynes were photographed before dissection. Vulvae were photographed after treating them in a 10% warm solution of potassium hydroxide (KOH) to dissolve soft tissues. Images were captured with a Canon EOS 750D wide zoom digital camera (24.2 megapixels) mounted on the stereomicroscope mentioned above and assembled using Helicon Focus v.3.10.3 image stacking software (Khmelik et al. 2005). Drawings were done with Procreate v.5.0.2 (Savage Interactive Pty. Ltd.). All measurements are given in millimeters (mm). Leg measurements are shown as total length (femur, patella, tibia, metatarsus and tarsus). Leg segments were measured on their dorsal sides. The distribution map was generated with ArcGIS v. 10.2 (ESRI Inc.). The specimens studied are preserved in 75% ethanol and deposited in the Institute of Zoology, Chinese Academy of Sciences (**IZCAS**) in Beijing, China.

Terminology and taxonomic descriptions follow Huber (2005) and Yao et al. (2015). The following abbreviations are used in the descriptions: **ALE** = anterior lateral eye, **AME** = anterior median eye, **PME** = posterior median eye, **L/d** = length/diameter; used in the illustrations: **aa** = anterior arch, **b** = bulb, **ba** = bulbal apophysis, **da** = distal apophysis, **e** = embolus, **ep** = epigynal pocket, **f** = flap, **pa** = proximo-lateral apophysis, **pp** = pore plate, **pr** = procursus.

## ﻿Taxonomy

### ﻿Family Pholcidae C.L. Koch, 1850

**Subfamily Pholcinae C.L. Koch, 1850**

#### ﻿Genus Belisana

Taxon classificationAnimaliaAraneaePholcidae

Thorell, 1898

71320D13-2C25-57B6-9E76-1897A17942D8

##### Type species.

*Belisanatauricornis* Thorell, 1898.

#### ﻿ Belisana langping

Taxon classificationAnimaliaAraneaePholcidae

Zhang, Li & Yao sp. nov.

1C08D9FE-3B6E-5004-AFA7-093BF1BC2DFD

https://zoobank.org/ED94621E-E599-4452-9CD0-7EFB7C35B999

**Figure 2.**
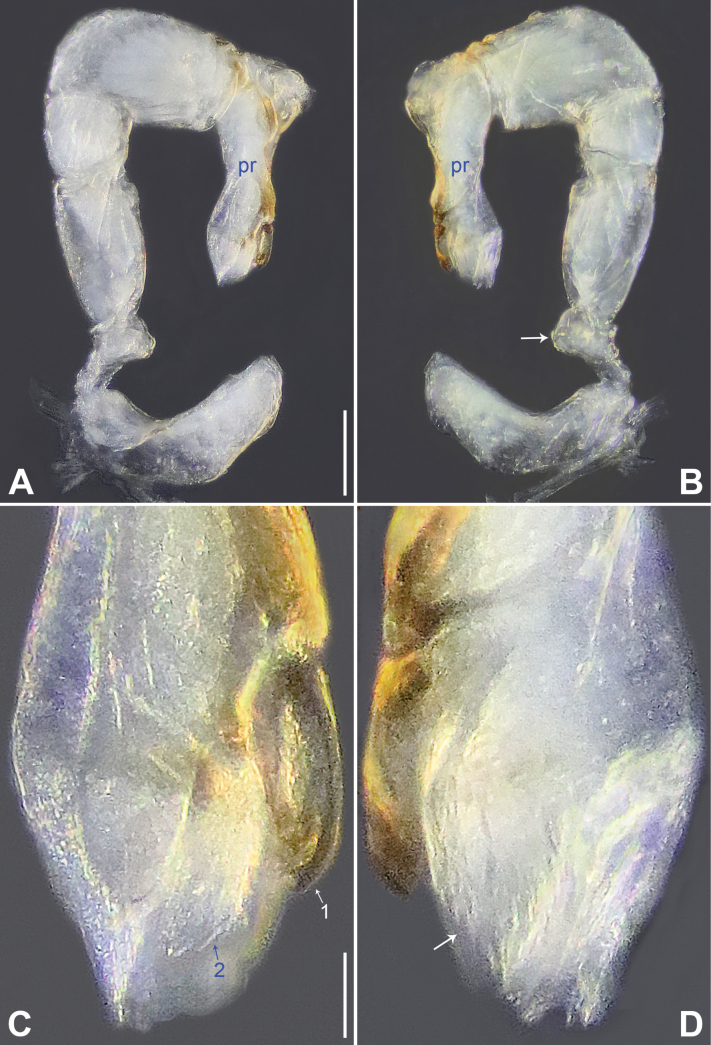
*Belisanalangping* sp. nov., holotype male **A, B** palp (**A** prolateral view **B** retrolateral view, arrow points at ventral apophysis) **C, D** distal part of procursus (**C** prolateral view, arrow 1 points at prolatero-subdistal apophysis, arrow 2 points at prolatero-subdistal membranous lamella **D** retrolateral view, arrow points at retrolatero-subdistal membranous lamella). Abbreviation: pr = procursus. Scale bars: 0.10 mm (**A, B**); 0.02 mm (**C, D**).

**Figure 3.**
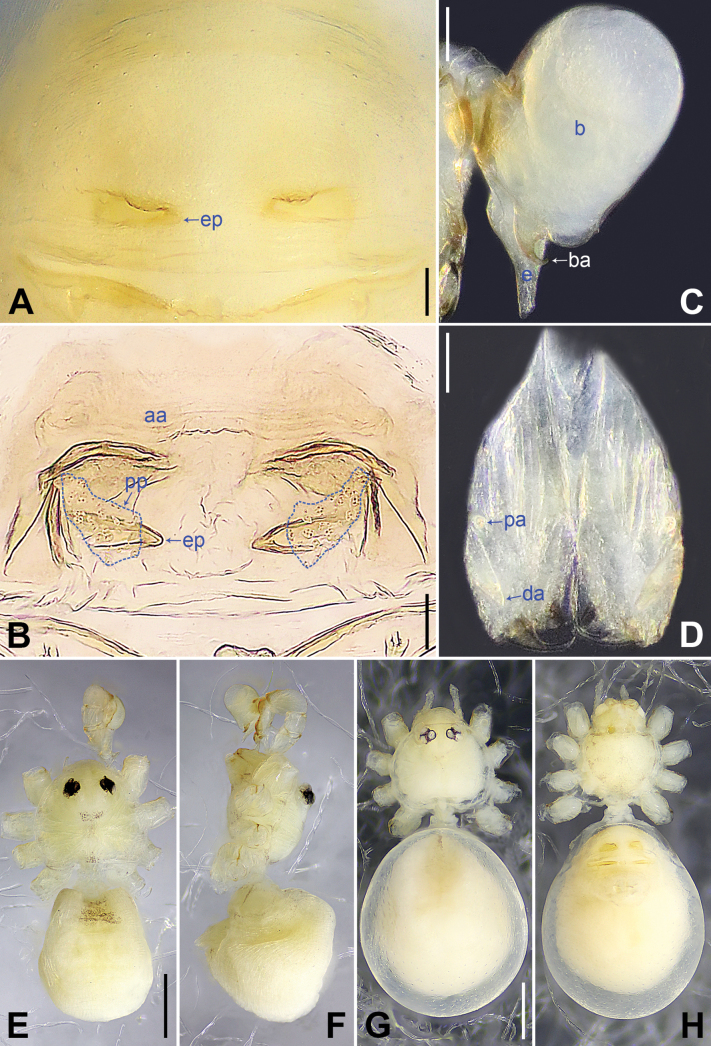
*Belisanalangping* sp. nov., holotype male (**C–F**) and paratype female (**A, B, G, H**) **A** epigyne, ventral view **B** vulva, dorsal view **C** bulb, prolateral view **D** chelicerae, frontal view **E–H** habitus (**E, G** dorsal view **F** lateral view **H** ventral view). Abbreviations: aa = anterior arch, b = bulb, ba = bulbal apophysis, da = distal apophysis, e = embolus, ep = epigynal pocket, pa = proximo-lateral apophysis, pp = pore plate. Scale bars: 0.05 mm (**A–D**); 0.30 mm (**E–H**).

Figs 2 , 3 , 8A, B , 9A, B

##### Type material.

***Holotype*: China** • ♂; Guangxi, Baise, Tianlin County, Langping Town, Dabao Village, Sanchuantun, Papa Cave; 24°34.226'N, 106°13.675'E; alt. 773 m; 14 Aug. 2011; C Wang leg.; IZCAS-Ar44988. ***Paratypes*: China** • 4♀; same data as for holotype; IZCAS-Ar44989–92.

##### Etymology.

The specific name refers to the type locality; noun in apposition.

##### Diagnosis.

The new species resembles *B.phungae* Yao, Pham & Li, 2015 (Yao et al. 2015: 9, figs 19A–D, 20A–G, 21A–E) by having similar male chelicerae and epigyne (Fig. 3A, D), but can be distinguished by procursus with retrolatero-subdistal membranous lamella (arrow in Figs 2D, 8B vs. absent in *B.phungae*), by bulbal apophysis hooked (ba in Fig. 3C vs. distally blunt in *B.phungae*), by cheliceral proximo-lateral apophyses and distal apophyses closer to each other (Fig. 3D vs. widely separated in *B.phungae*), by vulva without sac-like structure (Figs 3B, 9B vs. present in *B.phungae*), and by pore plates nearly triangular (pp in Figs 3B, 9B vs. nearly round in *B.phungae*); also distinguished from *B.zhangi* Tong & Li, 2007 (Tong and Li 2007: 505, figs 1–6) by procursus with sclerotized prolatero-subdistal apophysis (arrow 1 in Figs 2C, 8A vs. spine in *B.zhangi*), prolatero-subdistal membranous lamella (arrow 2 in Figs 2C, 8A vs. absent in *B.zhangi*), and retrolatero-subdistal membranous lamella (arrow in Figs 2D, 8B vs. retrolatero-ventral in *B.zhangi*), by procursus without retrolateral membranous flap (Figs 2D, 8B vs. present in *B.zhangi*), by vulval anterior arch straight (aa in Figs 3B, 9B vs. ridge-shaped in *B.zhangi*), and by pore plates nearly triangular (pp in Figs 3B, 9B vs. long and curved in *B.zhangi*).

##### Description.

**Male** (***holotype***): Total length 1.11 (1.20 with clypeus), prosoma 0.40 long, 0.53 wide, opisthosoma 0.71 long, 0.54 wide. Leg I missing, leg II: 4.88 (1.30, 0.20, 1.18, 1.62, 0.58), leg III: 3.92 (1.00, 0.19, 0.96, 1.27, 0.50), leg IV: 4.76 (1.27, 0.20, 1.17, 1.55, 0.57). Eye interdistances and diameters: PME–PME 0.10, PME 0.06, PME–ALE 0.02, AME absent. Sternum width/length: 0.43/0.33. Habitus as in Fig. 3E, F. Dorsal shield of prosoma yellowish, with indistinct median and posterior marks; sternum yellowish, with indistinct marginal marks. Legs whitish, without darker rings. Opisthosoma yellowish, without spots. Thoracic furrow absent. Clypeus unmodified. Chelicerae with pair of proximo-lateral apophyses (pa in Fig. 3D) and pair of distal apophyses (da in Fig. 3D; distance between tips: 0.11). Palp as in Fig. 2A, B; trochanter with ventral apophysis (arrow in Fig. 2B); procursus simple proximally but complex distally, with sclerotized prolatero-subdistal apophysis (arrow 1 in Figs 2C, 8A), prolatero-subdistal membranous lamella (arrow 2 in Figs 2C, 8A), and retrolatero-subdistal membranous lamella (arrow in Figs 2D, 8B); bulb with hooked apophysis (ba in Fig. 3C) and simple embolus (e in Fig. 3C).

**Female** (***paratype***, IZCAS-Ar44989): Similar to male, habitus as in Fig. 3G, H. Total length 1.57 (1.66 with clypeus), prosoma 0.43 long, 0.56 wide, opisthosoma 1.14 long, 0.90 wide. Leg I: 6.87 (1.86, 0.24, 1.78, 2.16, 0.83); tibia I L/d: 36. Eye interdistances and diameters: PME–PME 0.06, PME 0.05, PME–ALE 0.02, AME absent. Sternum width/length: 0.40/0.33. Epigyne simple and flat, with pair of median pockets 0.08 apart (ep in Figs 3A, 9A). Vulva with straight anterior arch (aa in Figs 3B, 9B) and pair of nearly triangular pore plates (pp in Figs 3B, 9B). Retrolateral trichobothria on tibia I at 5% proximally; legs with short vertical setae on metatarsi; tarsus I with 16 distinct pseudosegments.

##### Variation.

Tibia I in other three female paratypes (IZCAS-Ar44990–92): 1.80, 1.93, 1.98.

##### Habitat.

The species was found in the dark zone inside the cave.

##### Distribution.

China (Guangxi, type locality; Fig. 1).

#### ﻿ Belisana lingui

Taxon classificationAnimaliaAraneaePholcidae

Zhang, Li & Yao sp. nov.

1E9C3E64-934F-5342-823C-2185B1342211

https://zoobank.org/1B082AB3-F3BF-4B1D-B26E-E6E90BA19883

**Figure 4.**
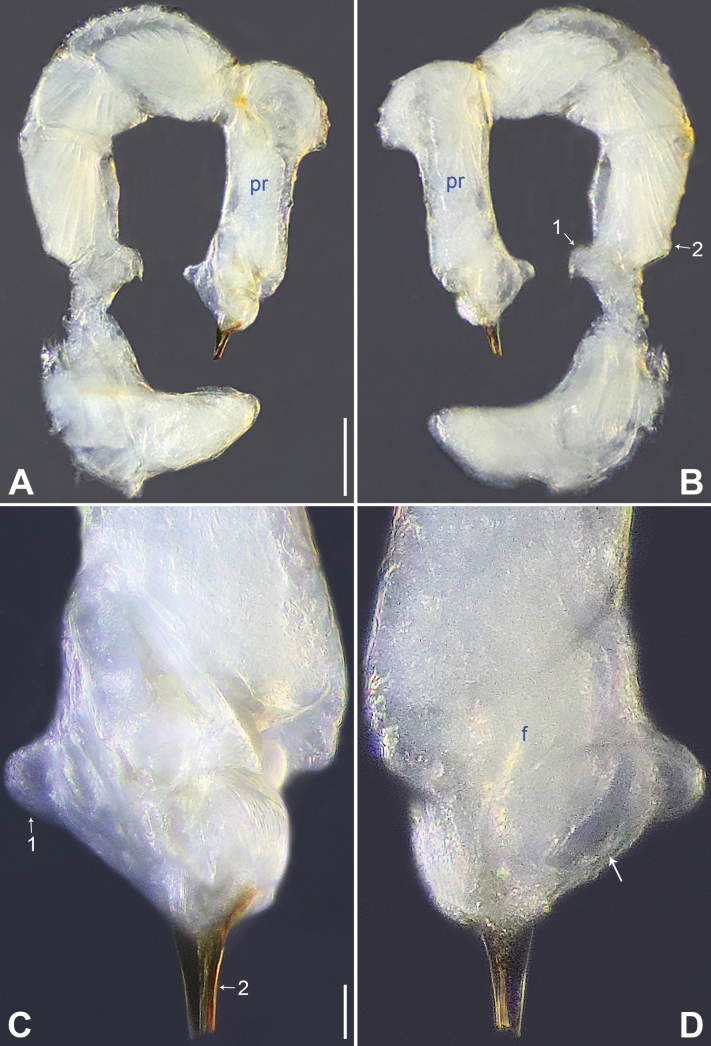
*Belisanalingui* sp. nov., holotype male **A, B** palp (**A** prolateral view **B** retrolateral view, arrow 1 points at ventral apophysis, arrow 2 points at retrolatero-proximal protrusion) **C, D** distal part of procursus (**C** prolateral view, arrow 1 points at ventro-subdistal membranous lamella, arrow 2 points at distal apophysis **D** retrolateral view, arrow points at retrolatero-subdistal membranous lamella). Abbreviations: f = flap, pr = procursus. Scale bars: 0.10 mm (**A, B**); 0.02 mm (**C, D**).

**Figure 5.**
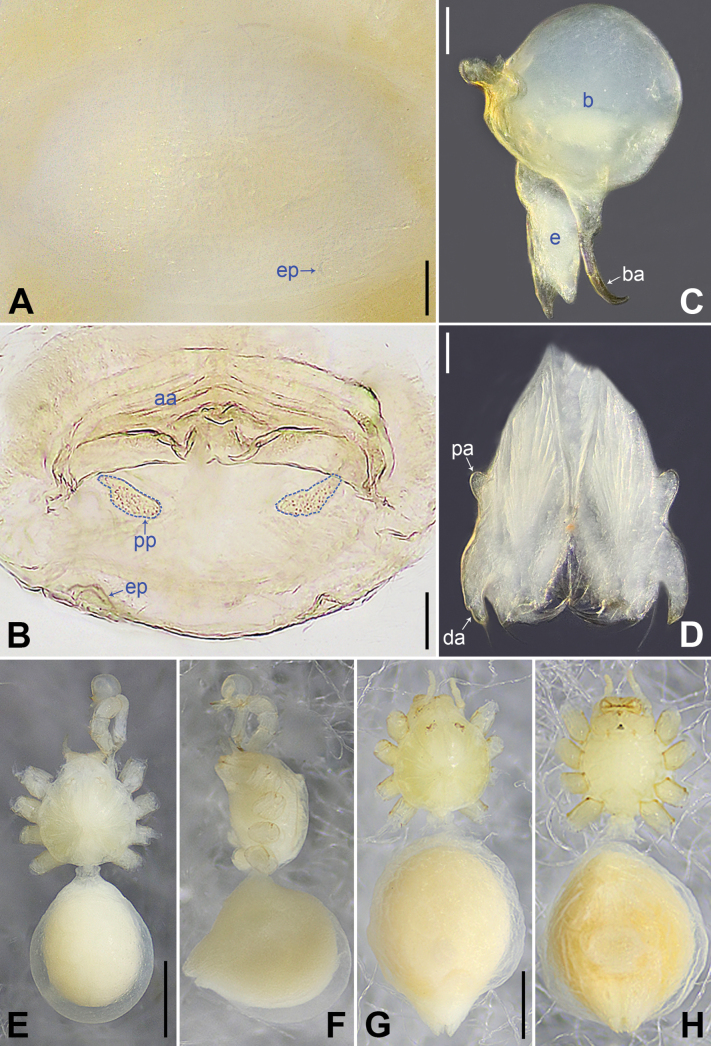
*Belisanalingui* sp. nov., holotype male (**C–F**) and paratype female (**A, B, G, H**) **A** epigyne, ventral view **B** vulva, dorsal view **C** bulb, prolateral view **D** chelicerae, frontal view **E–H** habitus (**E, G** dorsal view **F** lateral view **H** ventral view). Abbreviations: aa = anterior arch, b = bulb, ba = bulbal apophysis, da = distal apophysis, e = embolus, ep = epigynal pocket, pa = proximo-lateral apophysis, pp = pore plate. Scale bars: 0.05 mm (**A–D**); 0.30 mm (**E–H**).

Figs 4 , 5 , 8C, D , 9C, D

##### Type material.

***Holotype*: China** • ♂; Guangxi, Guilin, Lingui County, Yanmendi Village, Shuixianyan Cave; 25°12.819'N, 110°12.050'E; alt. 161 m; 18 Jul. 2009; Z Yao leg.; IZCAS-Ar44993. ***Paratypes*: China** • 3♀; same data as for holotype; IZCAS-Ar44994–96.

##### Etymology.

The specific name refers to the type locality; noun in apposition.

##### Diagnosis.

The new species resembles *B.galeiformis* Zhang & Peng, 2011 (Zhang and Peng 2011: 52, fig. 1A–F) by having similar bulbal apophyses and male chelicerae (Fig. 5C, D), but can be distinguished by procursus without prolatero-subdistal sclerite (Figs 4C, 8C vs. present in *B.galeiformis*), by epigyne with posterior pockets (ep in Figs 5A, 9C vs. median in *B.galeiformis*), by pore plates anteriorly narrow and posteriorly wide (pp in Figs 5B, 9D vs. elliptic in *B.galeiformis*), and by dorsal shield of prosoma without marks (Fig. 5E, G vs. with radiating marks in *B.galeiformis*); also distinguished from *B.tongle* Zhang, Chen & Zhu, 2008 (Zhang et al. 2008: 654, figs 1–5) by procursus without prolatero-subdistal sclerite and ventro-subdistal apophysis (Figs 4C, 8C vs. present in *B.tongle*).

##### Description.

**Male** (***holotype***): Total length 1.51 (1.59 with clypeus), prosoma 0.56 long, 0.59 wide, opisthosoma 0.95 long, 0.84 wide. Leg I: 20.66 (5.26, 0.33, 5.19, 7.98, 1.90), leg II: 14.16 (3.88, 0.33, 3.64, 5.00, 1.31), leg III: – (2.53, 0.30, 2.25, 3.00, –), leg IV: 11.97 (3.60, 0.30, 3.23, 3.76, 1.08); tibia I L/d: 80. Eye interdistances and diameters: PME–PME 0.12, PME 0.05, PME–ALE 0.02, AME absent. Sternum width/length: 0.49/0.44. Habitus as in Fig. 5E, F. Dorsal shield of prosoma and sternum yellowish, without marks. Legs whitish, without darker rings. Opisthosoma yellowish, without spots. Thoracic furrow absent. Clypeus unmodified. Eyes without pigments, but apparently with small lenses. Chelicerae with pair of proximo-lateral apophyses (pa in Fig. 5D) and pair of distal apophyses (da in Fig. 5D; distance between tips: 0.22). Palp as in Fig. 4A, B; trochanter with ventral apophysis (arrow 1 in Fig. 4B); femur with small retrolatero-proximal protrusion (arrow 2 in Fig. 4B); procursus simple proximally but complex distally, with ventro-subdistal membranous lamella (arrow 1 in Figs 4C, 8C), spine-shaped distal apophysis (tip broken; arrow 2 in Figs 4C, 8C), retrolatero-subdistal membranous lamella (arrow in Figs 4D, 8D), and retrolateral membranous flap (f in Figs 4D, 8D); bulb with hooked apophysis (ba in Fig. 5C) and simple embolus (e in Fig. 5C). Retrolateral trichobothria on tibia I at 4% proximally; legs with short vertical setae on metatarsi; tarsus I with 17 distinct pseudosegments.

**Female** (***paratype***, IZCAS-Ar44994): Similar to male, habitus as in Fig. 5G, H. Total length 2.00 (2.09 with clypeus), prosoma 0.58 long, 0.63 wide, opisthosoma 1.42 long, 1.01 wide; tibia I: 2.34; tibia I L/d: 39. Eye interdistances and diameters: PME–PME 0.11, PME 0.04, PME–ALE 0.02, AME absent. Sternum width/length: 0.50/0.44. Epigyne simple and flat, with pair of posterior pockets 0.20 apart (ep in Figs 5A, 9C). Vulva with ridge-shaped anterior arch (aa in Figs 5B, 9D) and pair of anteriorly narrow and posteriorly wide pore plates (pp in Figs 5B, 9D).

##### Variation.

Tibia I in the other two female paratypes (IZCAS-Ar44995–96): 2.34, 2.41.

##### Habitat.

The species was found in the dark zone inside the cave.

##### Distribution.

China (Guangxi, type locality; Fig. 1).

#### ﻿ Belisana tianyang

Taxon classificationAnimaliaAraneaePholcidae

Zhang, Li & Yao sp. nov.

370E6028-657B-58B5-B22C-14AC2195FE63

https://zoobank.org/4D5E7BF7-2704-4467-9EE5-056C1FFE1D33

**Figure 6.**
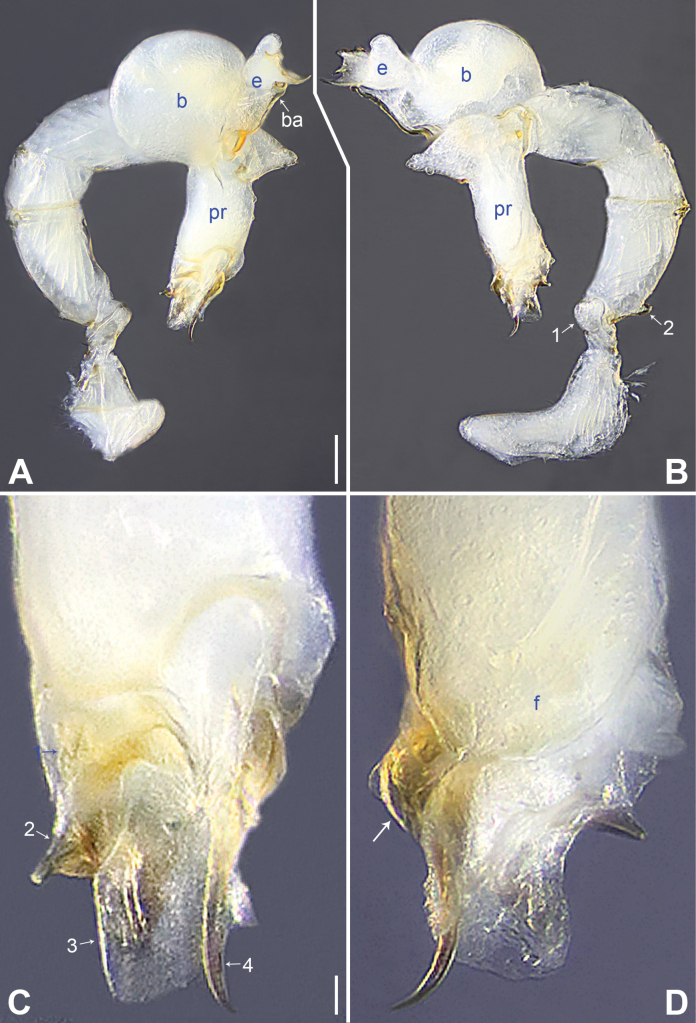
*Belisanatianyang* sp. nov., holotype male **A, B** palp (**A** prolateral view **B** retrolateral view, arrow 1 points at ventral apophysis, arrow 2 points at retrolatero-proximal protrusion) **C, D** distal part of procursus (**C** prolateral view, arrow 1 points at prolatero-subdistal sclerite, arrow 2 points at prolatero-ventral lamella, arrow 3 points at distal membranous lamella, arrow 4 points at distal spine **D** retrolateral view, arrow points at dorso-subdistal apophysis). Abbreviations: b = bulb, ba = bulbal apophysis, e = embolus, f = flap, pr = procursus. Scale bars: 0.10 mm (**A, B**); 0.02 mm (**C, D**).

**Figure 7.**
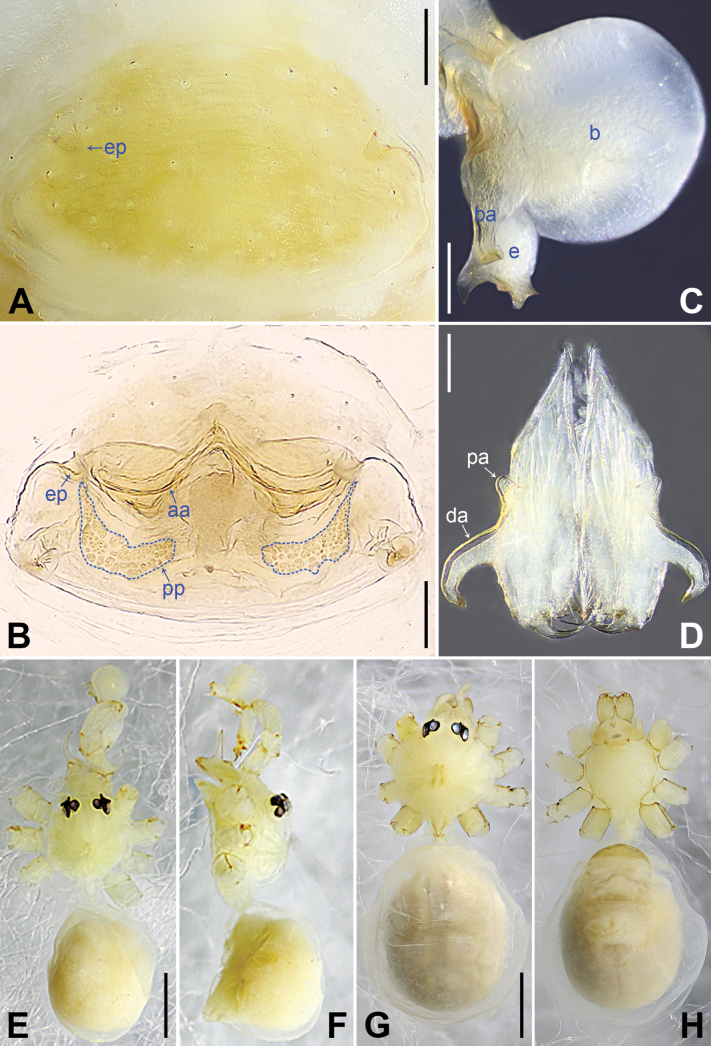
*Belisanatianyang* sp. nov., holotype male (**C–F**) and paratype female (**A, B, G, H**) **A** epigyne, ventral view **B** vulva, dorsal view **C** bulb, prolateral view **D** chelicerae, frontal view **E–H** habitus (**E, G** dorsal view **F** lateral view **H** ventral view). Abbreviations: aa = anterior arch, b = bulb, ba = bulbal apophysis, da = distal apophysis, e = embolus, ep = epigynal pocket, pa = proximo-lateral apophysis, pp = pore plate. Scale bars: 0.10 mm (**A–D**); 0.50 mm (**E–H**).

**Figure 8.**
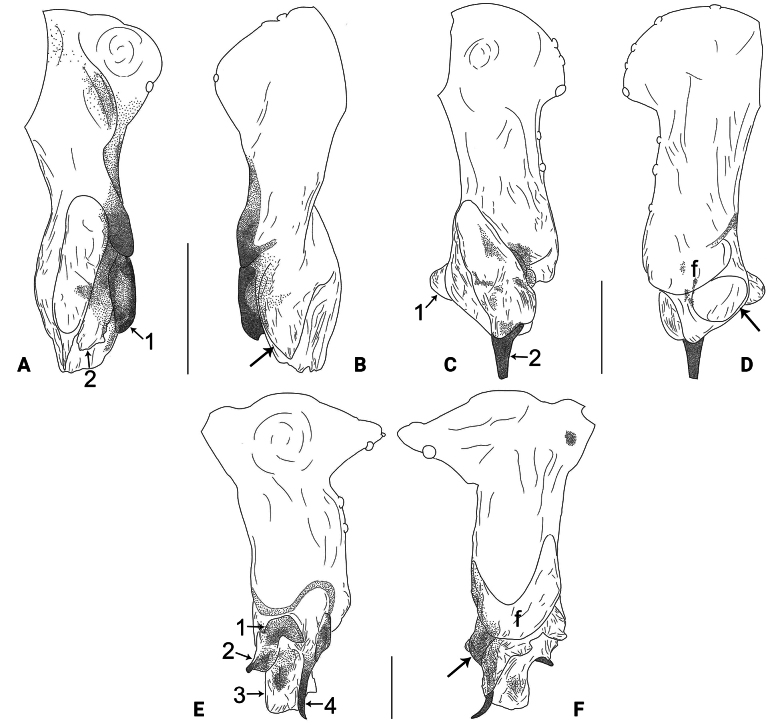
Procursus in prolateral and retrolateral views (arrows point at same structures as photos of each species) **A, B***Belisanalangping* sp. nov. **C, D***B.lingui* sp. nov. **E, F***B.tianyang* sp. nov. Abbreviation: f = flap. Scale bars: 0.10 mm.

**Figure 9.**
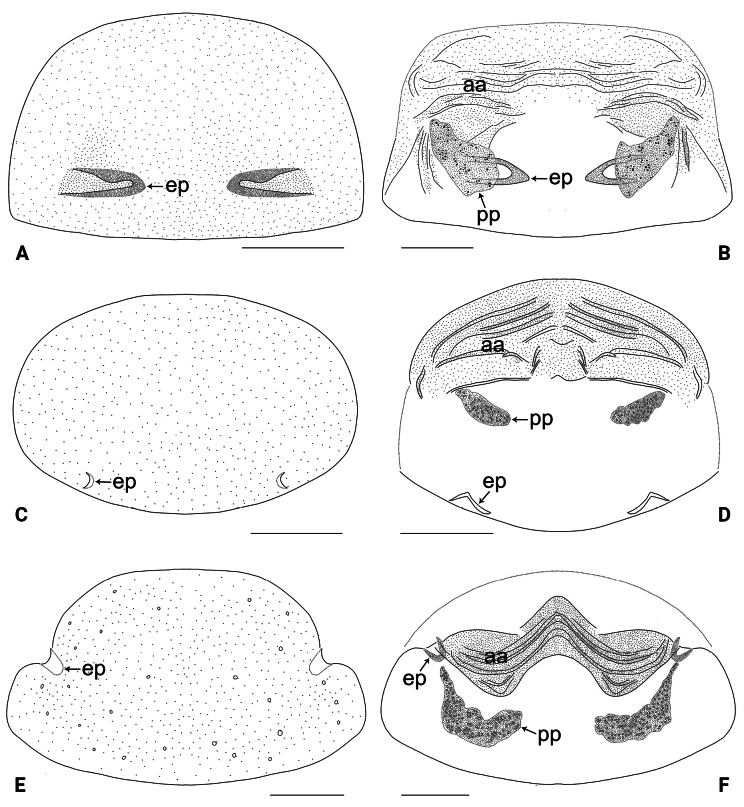
Female genitalia in ventral and dorsal views **A, B***Belisanalangping* sp. nov. **C, D***B.lingui* sp. nov. **E, F***B.tianyang* sp. nov. Abbreviations: aa = anterior arch, ep = epigynal pocket, pp = pore plate. Scale bars: 0.10 mm.

Figs 6 , 7 , 8E, F , 9E, F

##### Type material.

***Holotype*: China** • ♂; Guangxi, Baise, Tianyang County, Dongjing Town, Liangdongyan Cave; 23°40.123'N, 106°33.956'E; alt. 467 m; 7 Aug. 2011; C Wang leg.; IZCAS-Ar44997. ***Paratypes*: China** • 3♂; same data as for holotype; IZCAS-Ar44998–45000 • 2♀; same data as for holotype; IZCAS-Ar45001–02.

##### Etymology.

The specific name refers to the type locality; noun in apposition.

##### Diagnosis.

The new species resembles *B.tianlinensis* Zhang & Peng, 2011 (Zhang and Peng 2011: 65, fig. 10A–G) by having similar bulbal apophyses and epigyne (Fig. 7A, C), but can be distinguished by retrolateral flap of procursus strongly curved and wide (4 times wider than long, f in Figs 6D, 8F vs. straight and 2 times wider than long in *B.tianlinensis*), by male cheliceral distal apophyses long (6 times longer than wide) and tips widely separated (da in Fig. 7D vs. 2 times longer than wide and tips closer to each other in *B.tianlinensis*), by pore plates curved, anteriorly pointed and posteriorly wide (pp in Figs 7B, 9F vs. nearly triangular in *B.tianlinensis*), and by male clypeus unmodified (Fig. 7E vs. with pointed frontal apophysis in *B.tianlinensis*); also distinguished from *B.douqing* Chen, Zhang & Zhu, 2009 (Chen et al. 2009: 59, figs 1–11) by procursus with rectangular distal membranous lamella (arrow 3 in Figs 6C, 8E vs. nearly square in *B.douqing*) and curved retrolateral membranous flap (f in Figs 6D, 8F vs. angular in *B.douqing*) and by pore plates curved, anteriorly pointed and posteriorly wide (pp in Figs 7B, 9F vs. long elliptic in *B.douqing*).

##### Description.

**Male** (***holotype***): Total length 1.98 (2.08 with clypeus), prosoma 0.74 long, 0.75 wide, opisthosoma 1.24 long, 0.89 wide. Leg I: 21.95 (5.51, 0.33, 5.44, 9.17, 1.50), leg II missing, leg III: 8.82 (2.44, 0.27, 2.25, 3.13, 0.73), leg IV: 12.12 (3.60, 0.28, 3.04, 4.45, 0.75); tibia I L/d: 68. Eye interdistances and diameters: PME–PME 0.10, PME 0.08, PME–ALE 0.02, AME absent. Sternum width/length: 0.58/0.56. Habitus as in Fig. 7E, F. Dorsal shield of prosoma yellowish, with indistinct median stripe; sternum yellowish, without marks. Legs whitish, without darker rings. Opisthosoma yellowish, without spots. Thoracic furrow absent. Clypeus unmodified. Chelicerae with pair of proximo-lateral apophyses (pa in Fig. 7D) and pair of distal apophyses (da in Fig. 7D; distance between tips: 0.36). Palp as in Fig. 6A, B; trochanter with ventral apophysis (arrow 1 in Fig. 6B); femur with small retrolatero-proximal protrusion (arrow 2 in Fig. 6B); procursus simple proximally but complex distally, with prolatero-subdistal sclerite (arrow 1 in Figs 6C, 8E), sclerotized prolatero-ventral lamella (arrow 2 in Figs 6C, 8E), distal membranous lamella (arrow 3 in Figs 6C, 8E), curved distal spine (arrow 4 in Figs 6C, 8E), sclerotized dorso-subdistal apophysis (arrow in Figs 6D, 8F), and retrolateral membranous flap (f in Figs 6D, 8F); bulb with hooked apophysis (ba in Fig. 7C) and simple embolus (e in Fig. 7C). Retrolateral trichobothria on tibia I at 5% proximally; legs with short vertical setae on metatarsi; tarsus I with 19 distinct pseudosegments.

**Female** (***paratype***, IZCAS-Ar45001): Similar to male, habitus as in Fig. 7G, H. Total length 2.14 (2.27 with clypeus), prosoma 0.70 long, 0.79 wide, opisthosoma 1.44 long, 1.32 wide; tibia I: 4.15; tibia I L/d: 52. Eye interdistances and diameters: PME–PME 0.10, PME 0.08, PME–ALE 0.02, AME absent. Sternum width/length: 0.55/0.54. Epigyne simple and flat, with pair of lateral pockets 0.36 apart (ep in Figs 7A, 9E). Vulva with ridge-shaped anterior arch (aa in Figs 7B, 9F) and pair of curved, anteriorly pointed and posteriorly wide pore plates (pp in Figs 7B, 9F).

##### Variation.

Tibia I in three male paratypes (IZCAS-Ar44998–45000): 5.38, 5.64, 5.77. Tibia I in another female paratype (IZCAS-Ar45002) missing.

##### Habitat.

The species was found in the dark zone inside the cave.

##### Distribution.

China (Guangxi, type locality; Fig. 1).

## Supplementary Material

XML Treatment for Belisana

XML Treatment for Belisana langping

XML Treatment for Belisana lingui

XML Treatment for Belisana tianyang

## ﻿Additional information

### Author ORCIDs

Ludan Zhang https://orcid.org/0000-0001-5657-751X

Shuqiang Li https://orcid.org/0000-0002-3290-5416

Zhiyuan Yao https://orcid.org/0000-0002-1631-0949

### Data availability

All of the data that support the findings of this study are available in the main text.
